# The complete chloroplast genome of *Keteleeria evelyniana*

**DOI:** 10.1080/23802359.2021.1920488

**Published:** 2021-08-16

**Authors:** Yaqi Li, Jiangfei Li, Dan Wang, Yajin Zhu, Dawei Wang, Yulan Xu, Nianhui Cai

**Affiliations:** a Key Laboratory for Forest Resources Conservation and Utilization in the Southwest Mountains of China Ministry of Education, Southwest Forestry University, Kunming, China; b Key Laboratory for Forest Genetic and Tree Improvement and Propagation in Universities of Yunnan Province, Southwest Forestry University, Kunming, China

**Keywords:** *Keteleeria evelyniana*, chloroplast genome, phylogenetic analysis

## Abstract

Here, we report the complete chloroplast genome of *Keteleeria evelyniana*. The genome is 116,940 bp in size, which is comprised of a large single-copy (LSC) region of 74,075 bp, a small single-copy (SSC) region of 40,425 bp, and two short inverted repeat (IR) regions of 1,220 bp. The overall GC content of the plastome was 38.5%. The new sequence comprised 103 unique genes, including 74 protein-coding genes, 4 rRNA genes, and 25 tRNA genes. Phylogenetic analysis showed that *K.evelyniana* was close to *Keteleeria hainanensis* and *Keteleeria davidiana*.

*Keteleeria evelyniana* is an evergreen tree species belonging to the Pinaceae family, endemic to China. It is mainly distributed in Yunnan, Guizhou and Sichuan in China, occurring in altitude regions between 700 and 2600 m (He et al. 2011). It is not only a landscape tree species in Southwest China, but also an economically important tree species in Yunnan. It has been well demonstrated that *K.evelyniana* is a good material for furniture, due to its wood drying quickly, and easy to process (Zhang et al. 2008). Also, *K. evelyniana* is considered as useful materials for construction, bridges, wooden molds, ships, etc., due to its strong water resistance, moisture resistance and corrosion resistance. Additionally, this tree species is also considered as traditional Chinese medicine in China, that is associated with high medicinal value (Zhang et al. 2014).

The complete chloroplast genomes have been proven to be an effective biological tool for rapid and accurate species recognition as super-barcode (Chen et al. 2018; Yang et al. 2019). To date, many studies have performed whole chloroplast genome sequencing. However, for such an economically important tree species, the classification and phylogenetic relationships of *K. evelyniana* remain poorly known. In the present study, we report the complete chloroplast genome of *K. evelyniana*, thus providing useful information for future studies in genetic background and evolution.

The fresh leaves were collected from Southwest Forestry University Kunming, China. (Yunnan, China; geospatial coordinates: 102°45′41″E, 25°04′00″N; Altitude: 1945 m). The voucher specimens of *K. evelyniana* were deposited at the herbarium of Southwest Forestry University (Voucher number: SWFU-YS-0170) and DNA samples were stored at the Key Laboratory for Forest Resources Conservation and Utilization in the Southwest Mountains of China Ministry of Education, Southwest Forestry University, Kunming, China. The total genomic DNA was extracted by using the Magnetic beads plant genomic DNA preps Kit (TSINGKE Biological Technology, Beijing, China). A genomic shotgun library with an insert size of 350 bp was prepared and then this library was sequenced and 150 bp paired-end reads were generated. We assembled the chloroplast genome using GetOrganelle v1.6.2e (Jin et al. 2020). Genome annotation was performed with the online annotation tool DOGMA (Wyman et al. 2004) and program Geneious R8 (Kearse et al. 2012). Finally, the chloroplast DNA sequence with complete annotation information was submitted to GenBank with accession number MW043479.

The complete genome of *K. evelyniana* is 116,940 bp in size with a typical quadripartite structure, containing a small single-copy (SSC) region of 40,425 bp and a large single-copy (LSC) region of 74,075 bp separated by a pair of inverted repeat (IR) regions of 1220 bp. The total GC content is 38.5%, while the corresponding values of the LSC, IR, and SSC regions are 39.2%, 36.6%, and 37.4%, respectively. There were 103 unique genes, including 74 protein-coding genes, 4 rRNA genes, and 25 tRNA genes. Among them 12 genes (atpF, rpoC1, petB, petD, rpl16, rpl2, trnL-UAA, trnK-UUU, trnV-UAC, trnG-UCC, trnI-GAU, and trnA-UGC) have single introns, while one gene(ycf3) have double introns.

To explore the phylogenetic relationship of *K. evelyniana* in Pinaceae family, a maximum likelihood tree was constructed based on the complete chloroplast genomes of *K. evelyniana* and other 12 Pinaceae species. We aligned all chloroplast genomes by using MAFFT v7.307 (Katoh and Standley 2013) and analyzed by IQ-TREE 1.5.5 (Nguyen et al. 2015) under the TVM + F+R3 model. As illustrated in Figure 1, *K. evelyniana* appeared to be closely related to *Keteleeria hainanensis* and *Keteleeria davidiana*. The chloroplast genome of *K. evelyniana* will provide useful genetic information for further study on genetic diversity and conservation of Pinaceae species.

**Figure 1.**
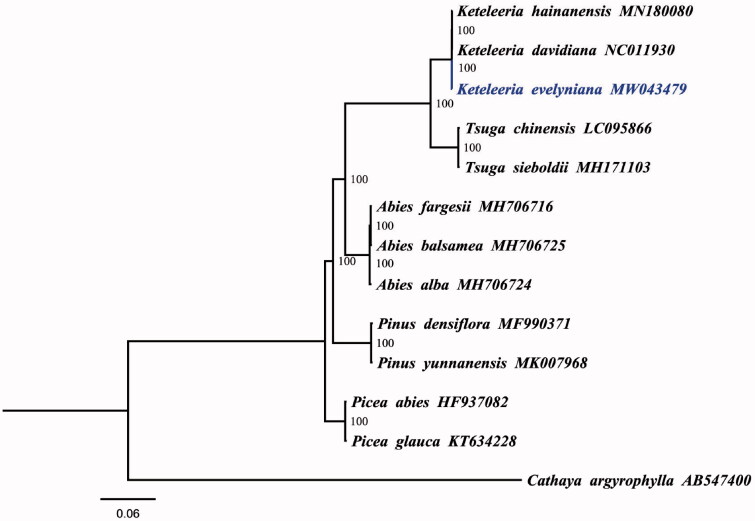
Maximum likelihood tree based on the complete chloroplast genome sequences of 13 Pinaceae species. The numbers on the branches are bootstrap values. GenBank accession numbers are shown in the figure.

## Data Availability

The data that support the findings of this study are openly available in GenBank at https://www.ncbi.nlm.nih.gov/genbank/, reference number: MW043479. The associated BioProject, SRA, and Bio-Sample numbers are PRJNA687643, SRR13354461, and SAMN17151149 respectively.
